# Electro-Fenton and Induced Electro-Fenton as Versatile Wastewater Treatment Processes for Decontamination and Nutrient Removal without Byproduct Formation

**DOI:** 10.1021/acsestengg.3c00128

**Published:** 2023-06-20

**Authors:** Luz Estefanny Quispe Cardenas, Parker John Deptula, Cynthia Soraya Huerta, Chonglin Zhu, Yinyin Ye, Siwen Wang, Yang Yang

**Affiliations:** † Department of Civil and Environmental Engineering, Clarkson University, Potsdam, New York 13699 United States; ‡ Institute for a Sustainable Environment, Clarkson University, Potsdam, New York 13699 United States; § Department of Civil, Structural and Environmental Engineering, University at Buffalo, Buffalo, New York 14260 United States

**Keywords:** electro-Fenton, kinetic model, radical chemistry, water treatment, disinfection

## Abstract

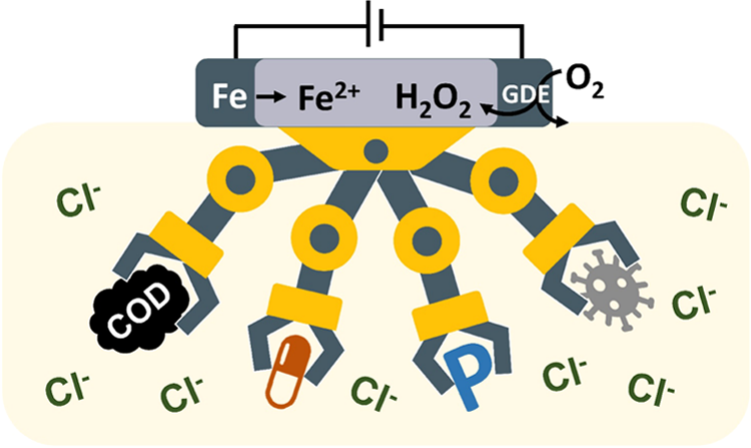

It is a long-pursued goal to develop electrified water treatment technology that can remove contaminants without byproduct formation. This study unveiled the overlooked multifunctionality of electro-Fenton (EF) and induced EF (I-EF) processes to remove organics, pathogens, and phosphate in one step without halogenated byproduct formation. The EF and I-EF processes used a sacrificial anode or an induced electrode to generate Fe^2+^ to activate H_2_O_2_ produced from a gas diffusion cathode fed by naturally diffused air. We used experimental and kinetic modeling approaches to illustrate that the ^•^OH generation and radical speciation during EF were not impacted by chloride. More importantly, reactive chlorine species were quenched by H_2_O_2_, which eliminated the formation of halogenated byproducts. When applied in treating septic wastewater, the EF process removed >80% COD, >50% carbamazepine (as representative trace organics), and >99% phosphate at a low energy consumption of 0.37 Wh/L. The EF process also demonstrated broad-spectrum disinfection activities in removing and inactivating *Escherichia coli*, *Enterococcus durans*, and model viruses MS2 and Phi6. In contrast to electrochemical oxidation (EO) that yielded mg/L level byproducts to achieve the same degree of treatment, EF did not generate byproducts (chlorate, perchlorate, trihalomethanes, and haloacetic acids). The I-EF carried over all the advantages of EF and exhibited even faster kinetics in disinfection and carbamazepine removal with 50–80% less sludge production. Last, using septic wastewater treatment as a technical niche, we demonstrated that iron sludge formation is predictable and manageable, clearing roadblocks toward on-site water treatment applications.

## Introduction

On-site wastewater treatment systems (OWTS) are critical to supplement the centralized wastewater treatment plants. The on-site systems serve rural areas that sewage pipelines cannot reach. Septic tanks, as the most mature OWTS, are used by more than 25% of the population in the United States.^1^ A functioning septic system can remove ∼50% of biochemical oxygen demand and 80% of total suspended solids,^2,3^ which substantially reduces the pollutant discharge to surface and groundwater. However, the septic system is less effective for removing pharmaceuticals and personal care products (PPCPs).^4,5^ Many reports also revealed the viral and bacterial contamination of surface and groundwater from the septic system.^6,7^ Last, septic tank effluents with high phosphorus concentration were identified as the primary contributor to algae growth in lakes, where phosphorus is usually the limiting nutrient.^8−10^

We acknowledge the critical role of conventional OWTS systems (i.e., septic tanks) in reducing regional contaminant discharge. However, innovations to address the intertwined challenges mentioned above represent an urgent need. Electrochemical oxidation (EO) treatment of sewage was extensively studied.^11,12^ Although EO could remove chemical oxygen demand and pathogen inactivation, the formation of byproducts is a significant concern.^13,14^ Hydrogen peroxide (H_2_O_2_)-driven advanced oxidation processes (UV/H_2_O_2_, Fenton, O_3_/H_2_O_2_, *etc*.) have long been considered promising solutions for centralized and distributed wastewater treatment.^15^ Among them, the Fenton-based reaction suits sewage treatment with poor UV transmittance and high ozone demand.

A typical Fenton reaction uses Fe^2+^ and H_2_O_2_ as the catalyst and radical reservoir, respectively. A major limitation of Fenton-based reactions is their stringent requirement for low pH to prevent the hydrolysis and oxidation of Fe^2+^ from forming iron hydroxides (Fe(OH)*_x_*) sludge.^16,17^ In order to operate at neutral pH, many advanced heterogeneous catalysts were developed to expose active sites without metal leaching.^18−20^ These fundamental breakthroughs deeply advanced our understanding of the Fenton reaction mechanism and diversified the technical options. However, from an engineering point of view, it is clear that Fe^2+^ is still the most affordable and accessible catalyst. Although they are deemed undesired products, the iron species (Fe^2+^, Fe^3+^, Fe(OH)*_x_*) may promote the removal of particulate chemical oxygen demand (COD) and pathogens by coagulation and benefit phosphorus sequestration.

Recently, various chemical-free electro-Fenton (EF) processes were developed, featuring the on-site generation of H_2_O_2_ (via cathodic H_2_O_2_ production by oxygen reduction) and Fe^2+^ (by the corrosion of iron electrodes).^16,21^ Most prior research activities reported process innovations for removing model pollutants.^22^ A system-level evaluation of the performance of EF-derived systems on several wastewater treatment aspects under real-world application scenarios is critically needed. The advantages and limitations of EF-like technologies compared with other electrified processes are yet to be disclosed.

We recently developed a highly efficient gas diffusion electrode (GDE) for the cathodic H_2_O_2_ electrosynthesis.^23^ This study further explores the facile activation of electro-synthesized H_2_O_2_ by sacrificial or induced iron electrodes. By unbiasedly comparing their performance with mainstream electrified water treatment processes, we demonstrated the multifunctional capabilities of EF and induced EF (I-EF) in the energy-effective simultaneous removal of bulk organics, micropollutants, pathogens, and phosphate in septic wastewater. Quantitative strategies for iron sludge management were provided. Moreover, via holistic experimental and simulative approaches, we identified the pivotal role of H_2_O_2_ in quenching chlorine, thereby eliminating the formation of byproducts. The findings accomplished a long-sought goal-a chemical-free electrified water treatment technology without byproduct formation.

## Experimental Procedure

### Chemicals and Materials

Potassium titanium oxide oxalate dihydrate (K_2_[TiO(C_2_O_4_)_2_·2H_2_O]), concentrated nitric acid (HNO_3_), concentrated sulfuric acid (H_2_SO_4_), and sodium perchlorate (NaClO_4_) were obtained from Fisher Scientific. Benzoic acid (**BA**), carbamazepine (**CMZ**), and hydroxylamine hydrochloride were purchased from Thermo Scientific. O-phenanthroline was obtained from LabChem. Inc.

The materials for the preparation of composite GDE cathode were described previously.^23^ The low-carbon steel (**LCS**) plate (carbon content 0.13–0.20%; 6 cm^2^) was purchased from McMaster-Carr. Ni–Sb–SnO_2_ anode (**NATO**; 6 cm^2^) was prepared following Zhang et al.^24^ Dimensionally stable iridium oxide (IrO*_x_*; 6 cm^2^) anode was purchased from Entrustech, China. Septic wastewater was collected from a septic tank of a family of three in Potsdam, NY. Water characteristics can be found in Table S1.

### Construction of Five Electrified Water Treatment Processes

**Figure 1:**
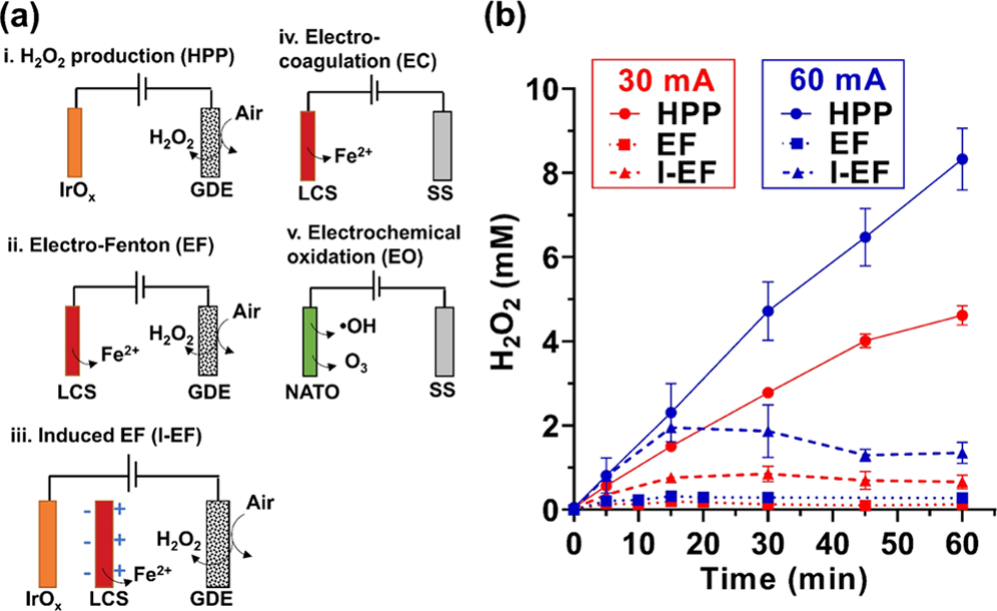
(a) Configurations of electrolytic cells investigated in this study. (b) Evolution of H_2_O_2_ in various modes. The areas of all electrodes are 6 cm^2^, except 12.5 cm^2^ for GDE. The electrolyte is 10 mM NaClO_4_ (60 mL) at pH = 6.5.

Figure 1a shows the configurations of five electrified water treatment processes investigated in this study. The spacing between the anode and cathode was 4 cm. The GDE cathode (12.5 cm^2^) was installed on one side of the single-chamber electrolytic cell (60 mL) with an open window for air diffusion. Coupling GDE with an IrO*_x_* anode enables H_2_O_2_ production (**HPP**). Pairing the LCS anode (6 cm^2^) with the GDE cathode drives the EF reactions. Placing a piece of LCS plate (6 cm^2^) between the IrO*_x_* anode and the GDE cathode constituted the induced EF (**I-EF**) process. The LCS plate was not connected to the electric circuit but was exposed to the electric gradient between the anode and cathode. Induced negative and positive potentials were created on the two sides of the LCS plate facing the IrO*_x_* anode and GDE cathode, respectively. Consequently, anodic corrosion released Fe^2+^ that catalyzed the Fenton reactions.^25,26^

For the investigation of the electrocoagulation (**EC**) process, a stainless-steel (**SS**) cathode (6 cm^2^) was paired with an LCS anode (6 cm^2^). For the electrochemical oxidation (**EO**) process, the Ni–Sb–SnO_2_ anode (**NATO**; 6 cm^2^) was coupled with a SS cathode. The NATO anode was selected because of its superior performance in organic destruction in sewage wastewater, outperforming other commercial anodes, including boron-doped diamond and IrO*_x_* electrodes.^24^

### Sampling and Analysis

Water samples generated in EF, I-EF, and EC processes are a mixture of liquid and iron sludge, denoted as mixed liquor. Supernatant samples were obtained by centrifuging mixed liquor at 3500 rpm for 1 min. The analysis of mixed liquor characterizes the treatment efficiency by oxidation alone, as the contaminants entrapped in flocs were also quantified. The analysis of supernatant after liquid/solid separation evaluates the removal contributed jointly by oxidation and coagulation.

H_2_O_2_ was quantified by the potassium titanium oxalate spectrophotometric method.^27^ Fe^2+^ was quantified by the o-phenanthroline method.^28^ To measure total dissolved iron (Fe^2+^ and Fe^3+^), iron species were reduced to Fe^2+^ using hydroxylamine hydrochloride. The COD was analyzed following the HACH method 8000. The differentiation of COD removal by coagulation and oxidation was calculated based on an adapted experimental method by Han et al.,^29^ as detailed in Text S1. BA and CMZ were analyzed by an ultra-high performance liquid chromatography system (ExionLC 2.0+) coupled with a quadrupole time-of-flight mass spectrometer (SCIEX 5600 Model X500B). Details are available in Texts S2 and S3. Trihalomethanes (**THMs**) and haloacetic acids (**HAAs**) were analyzed by gas chromatography/mass spectrometry.^13^ Anions, including chloride, chlorate, perchlorate, and phosphate, were analyzed by ion chromatography (Dionex).

### Microbial Cultivation and Analysis

*Escherichia coli* (*E. coli* K-12, ATCC 10798) and *Enterococcus durans* (*E. durans*, ATCC 6056) were selected as model gram-negative and gram-positive bacteria, respectively. These bacteria were commonly used as non-pathogenic surrogates of other disease-causing bacteria in wastewater disinfection.^24,30,31^ Two bacteriophages were selected as surrogates of various infectious viruses:^30,32,33^ MS2 (ssRNA; non-enveloped; ATCC 15597-B1) and Phi6 (dsRNA; enveloped; provided by Dr Ye of the University at Buffalo). The bacterial hosts, *E. coli* C3000 (ATCC 15597) for MS2 and *Pseudomonas psyringae* for Phi6, were used for virus propagation and plaque essays for virus titers. To investigate disinfection efficiency, model bacteria and viruses were spiked in sterilized septic wastewater to final concentrations of ∼10^5^ to 10^6^ CFU/mL or PFU/mL. More details on cultivation, sampling, and analysis are available in Text S4.

## Results and Discussion

### Electrochemical H_2_O_2_ Production and Activation

The electrosynthesis of H_2_O_2_ from oxygen in the air (Rxn 1) was realized by a composite GDE, which contains a layer of electrospun PTFE fibers on top of carbon black catalysts loaded on carbon paper.^23^ We have conducted a holistic investigation to show that the multilayer configuration prohibits the cathodic decomposition of as-formed H_2_O_2_. It enables the production of H_2_O_2_ at >85% faradaic efficiency in a wide current density window (2–20 mA/cm^2^) using air diffused from the air-facing side of GDE as the oxygen source.^23^

In this study, we first quantified the production of H_2_O_2_ in HPP mode by coupling the GDE cathode with the IrO*_x_* anode. Based on the H_2_O_2_ evolution rate measured in the initial 20 min, the current efficiency is calculated as 91 and 88% at 30 and 60 mA, respectively (Figure 1b). To drive the EF process, GDE was paired with LCS sacrificial anode. Under anodic potentials, the LCS was corroded to release ferrous (Fe^2+^) and ferric (Fe^3+^) ions (Rxns 2 and 3), which served as Fenton catalysts to convert H_2_O_2_ to ^•^OH (Rxn 4) or HO_2_^•^ (Rxn 5),^16^ evidenced by the negligible [H_2_O_2_] compared with the HPP mode.
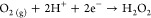
Rxn 1
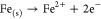
Rxn 2
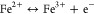
Rxn 3
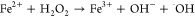
Rxn 4
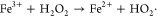
Rxn 5Conventional Fenton reactions require acidic conditions (pH <3) to maintain the availability of soluble Fe^2+^ catalysts.^16^ At neutral pH and above, Fe^2+^ will be hydrolyzed to Fe(OH)*_x_* or yield ferryl ions rather than radicals.^34^ In contrast, the chemical-free EF process can be operated within a wide pH window due to the continuous production of Fe^2+^. As shown in Figure S1, [Fe^2+^] at mg/L levels was maintained throughout the EF reaction in synthetic electrolytes (pH = 6.5) and septic wastewater (pH = 8). The I-EF process generated fewer Fe(OH)*_x_* than the EF process, leading to higher residual [H_2_O_2_] throughout the treatment (Figure 1b). This feature has several advantages in organic removals, disinfection, and sludge management, as discussed in the following sections.

**Figure 2:**
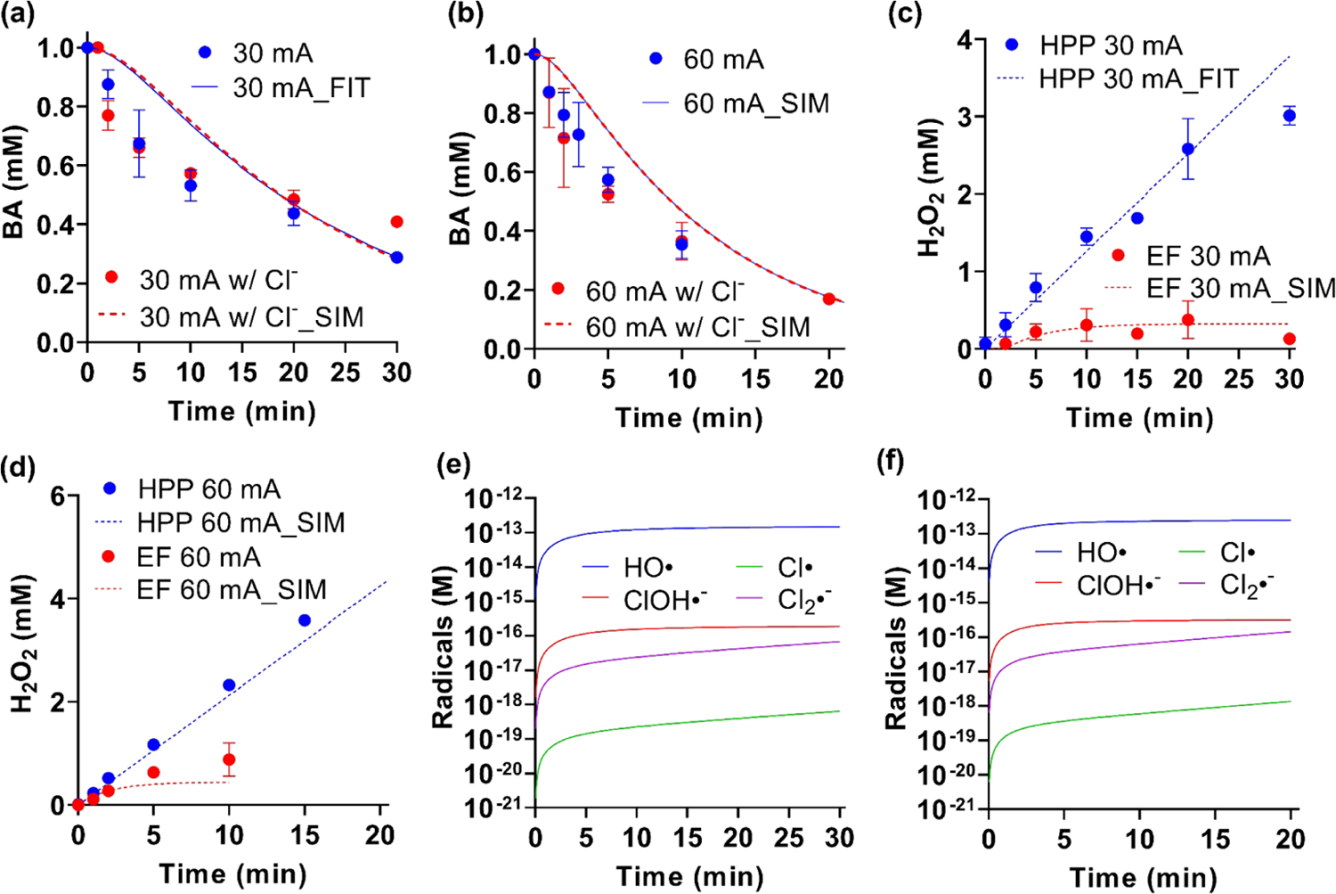
EF degradation of 1 mM BA in 10 mM NaClO_4_ (60 mL; pH = 4.0) in the absence or presence of 1.8 mM Cl^–^ at (a) 30 mA and (b) 60 mA in EF mode. H_2_O_2_ evolution in HPP and EF processes at (c) 30 and (d) 60 mA. At both currents, the potentials of the IrO*_x_* anode for HPP mode and LCS anode for EF mode were stabilized at ∼1.5 and ∼0.9 V_RHE_, respectively. Model simulation on radical speciation at (e) 30 and (f) 60 mA when 1.8 mM Cl^–^ was present. Dots are experimental data, while lines are modeling results. The data set denoted by “FIT” means the data were fed to the kinetic models to calibrate specific rate constants; those tagged as “SIM” are results predicted by the calibrated kinetic models without manual intervention.

Both EF and I-EF processes can convert H_2_O_2_ to ^•^OH. We first used BA, a chemical reactive toward ^•^OH (5.8 × 10^9^ M^–1^ s^–1^),^35^ to probe the ^•^OH production in the EF process. The control tests indicated that BA could not be removed by the EC process (Figure S2). Degradation of BA was only observed in the EF reactions, of which kinetics positively correlated with applied current (Figure 2a,b). The direct electron transfer oxidation of BA, which was only observed at anodic potentials >2.5 V versus reversible hydrogen electrode (V_RHE_) on boron-doped diamond electrodes,^36^ should not occur in EF mode with LCS anode potential at 0.9 V_RHE_. Therefore, the decay of BA in EF mode was solely attributed to reactions with radicals.

Chloride (Cl^–^), a ubiquitous component in wastewater, impacts the radical speciation profiles, changing the dominant radical species from ^•^OH to less oxidative Cl^•^ and Cl_2_^•–^.^11,37^ The septic tank water we aimed to treat contained 1.8 mM Cl^–^ (Table S1). Therefore, an equal amount of Cl^–^ was spiked into the synthetic electrolyte to elucidate its role in the EF process. When Cl^–^ exists, BA can react with not only ^•^OH but also with Cl^•^ and Cl_2_^•–^ (*k*_Cl_^•^ = 1.8 × 10^10^ M^–1^ s^–1^, *k*_Cl2_^•–^ = 2 × 10^6^ M^–1^ s^–1^).^35,38,39^ The degradation kinetics should differ from the ^•^OH-only scenario. However, this was not the case in our observation, as the degradation kinetics of BA in the EF process was not affected by Cl^–^ (Figure 2a,b).

To explain the impact of Cl^–^ on the EF process, we developed a comprehensive kinetic model that contains 47 pivotal reactions for H_2_O_2_ generation (Rxn S1 in Table S2), activation to ^•^OH (Rxn S2), radical transformation (Rxns S24–S27), BA degradation (Rxns S39–S42), quenching of oxidants and radicals by H_2_O_2_ (Rxns S43–46), and parallel chlorine evolution by IrO*_x_* anode in the HPP process (Rxn S47). The model solving was assisted by the Kintecus software.^40^ The rate constants of Rxns S1 and S2 are fitted by the data of the H_2_O_2_ production in the HPP process and the BA degradation without Cl^–^ in the EF process at 30 mA, respectively (data set marked as “FIT”). For processes at 60 mA, the rate constants of Rxns S1 and S2 were doubled, assuming the reaction rates were proportional to the current (i.e., electron flux).

Model accuracy was validated by successfully predicting the residual [H_2_O_2_] in the HPP and EF processes without Cl^–^ (the “SIM” data set in Figure 2c,d). After calibrating the rate of Rxn S47 (Cl^–^ + H_2_O → OCl^–^ + 2e^–^ + 2H^+^) catalyzed by IrO*_x_* anode, the model could reflect the experimental results that the presence of chloride slightly retarded H_2_O_2_ production in HPP mode (Figure S3). Additionally, no free chlorine (HClO/OCl^–^) was detected in the HPP process. Therefore, it is concluded that, though chlorine could be produced by the IrO*_x_* anode, it was readily quenched by the cathodically generated H_2_O_2_ (Rxns S45 and S46). As for the EF process, both modeling results and experimental data suggest that Cl^–^ did not impact the [H_2_O_2_] profiles (Figure S3). This is because the anodic reaction was dominated by LCS corrosion and could not oxidize chloride to chlorine to react with H_2_O_2_ (i.e., Rxn S47 can be excluded).

The kinetic model also provides critical insights into radical speciation. Assuming that ^•^OH is the only radical species in the absence of Cl^–^, the steady-state ^•^OH concentration ([^•^OH]_ss_) can be calculated by transforming a bimolecular reaction into a pseudo-first-order reaction.
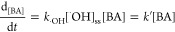
1
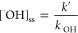
2where *k*′ is the pseudo-first-order rate constant obtained by fitting experimental data in Figure 2a,b by first-order reaction kinetics. The [^•^OH]_ss_ are calculated as 1.3 × 10^–13^ and 3.1 × 10^–13^ mol/L at 30 and 60 mA, respectively. The model simulation of radical profiles during EF reactions gives comparable values of 1.5 × 10^–13^ for 30 mA and 2.5 × 10^–13^ mol/L for 60 mA (Figure S4).

We further simulated the radical speciation when Cl^–^ exists. As shown in Figure 2e,f, ^•^OH is still the dominant radical. These features differ significantly from the EO process, where Cl^•^ and Cl_2_^•–^ should be the dominant species in treating Cl^–^ containing solutions.^11,37^ The model simulation indicates that Cl^•^ + H_2_O_2_ → HO_2_^•^ + Cl^–^ + H^+^ (Rxn S44) is the critical elementary reaction that prohibits the formation of Cl^•^ and Cl_2_^•–^. If this reaction is excluded, the Cl_2_^•–^ becomes the dominant species (Figure S5). Combining experimental and modeling approaches implies that the EF generated abundant ^•^OH as the dominant radical species. Such patterns were not altered in the presence of Cl^–^. Finally, the model simulation suggested that Cl^–^ did not impact BA degradation (Figure 2a,b), which reflected the experimental data. Given the above, it is concluded that, in addition to serving as a radical reservoir, H_2_O_2_ quenched reactive chlorine species (Cl^•^, Cl_2_^•–^, HClO/OCl^–^). Therefore, the ^•^OH evolution profile and BA decay kinetics were not impacted by Cl^–^ in the EF process.

**Figure 3:**
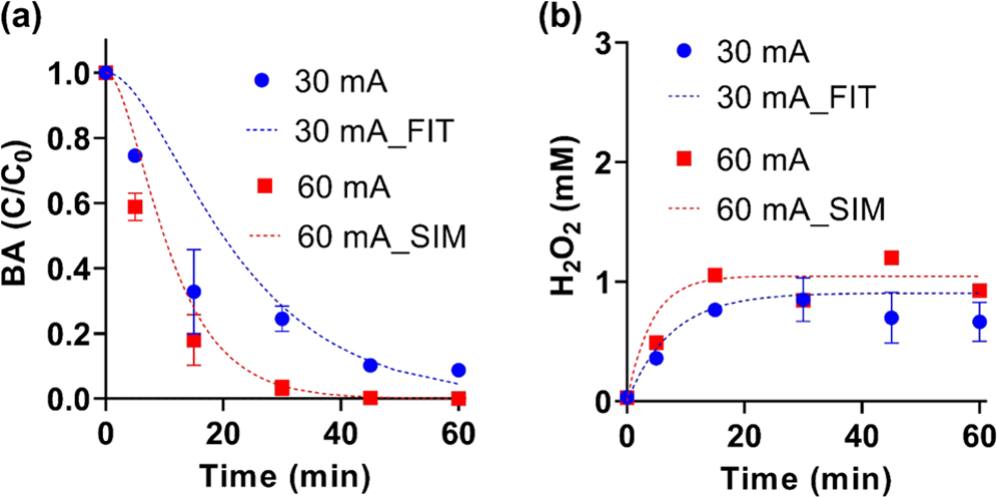
(a) Decay of 1 mM BA and (b) evolution of H_2_O_2_ in 10 mM NaClO_4_ (60 mL; pH = 4.0) in the I-EF process. Dots are experimental data, while lines are modeling results. The data set denoted by “FIT” means the data were fed to the kinetic models to calibrate specific rate constants; those tagged as “SIM” are results predicted by the calibrated kinetic models without manual intervention.

The BA degradation profiles of the I-EF process largely overlap with those of the EF process (Figures 3a and S2), implying the same level of [^•^OH]_ss_ was produced. The EF kinetic models were compatible with the I-EF process after recalibrating the rate constants of H_2_O_2_ activation (Rxn S2) and ^•^OH annihilation (Rxn S27) by experimental data at 30 mA. The calibrated model then successfully predicted the BA degradation and H_2_O_2_ evolution profiles at 60 mA (Figure 3a,b). The rate constants of Rxn S2 and S27 of I-EF are smaller than that of EF, which might be explained as less Fe^2+^ released by the induced current led to a slower H_2_O_2_ activation but also minimized the loss of ^•^OH (for example, Fe^2+^ + ^•^OH → Fe^3+^ + OH^–21^). These two mechanisms jointly contributed to the comparable BA degradation kinetics (i.e., [^•^OH]_ss_ levels) with the EF process. Since the I-EF process yielded a higher residual [H_2_O_2_] than the EF process (Figures 1b and 3b), it is reasonable to conclude that reactive chlorine species were also readily quenched, leaving ^•^OH the dominant oxidant.

### Removal of Organics and Phosphate in Septic Wastewater

After unraveling the radical chemistry in the synthetic electrolyte, the investigation was expanded to septic wastewater treatment. The wastewater collected from a household septic tank has a pH of 8.0 and conductivity of 997 μS/cm. Components included COD (307 mg/L), NH_4_^+^ (53 mg/L), total phosphate (3.0 mg/L), and Cl^–^ (64 mg/L). We performed unbiased comparisons of the treatment performance of EF, I-EF, EO, and EC (the HPP process did not exhibit any efficacy in COD removal and therefore was not discussed). All of the experiments were performed at 30 mA current to treat 60 mL of wastewater. The electrodes or induced LCS plate areas were kept at 6 cm^2^ except for GDE at 12.5 cm^2^.

**Figure 4:**
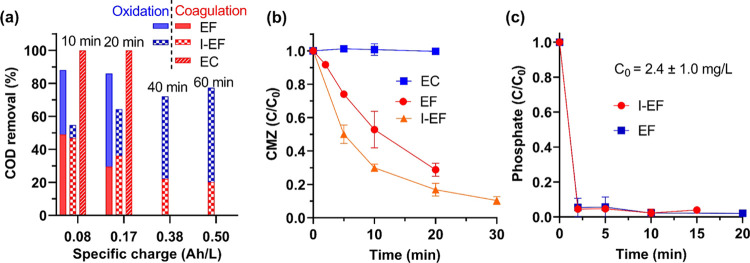
(a) COD removal in septic wastewater by EF, I-EF, and EC processes. Contributions of oxidation and coagulation to COD removal were marked in blue and red, respectively. Time marks represent the residence times. (b) Removal of CMZ (C_0_ = 1 μM) spiked in septic wastewater by EF, I-EF, and EC. (c) Phosphate removal in septic wastewater by EF and I-EF. All experiments were performed at 30 mA. The surface areas of IrO*_x_* anodes (in HPP and I-EF), induced LCS plate (I-EF), LCS anode (EC), and SS cathode (EC) were kept at 6 cm^2^. GDE cathode used in EF and I-EF has an area of 12.5 cm^2^. The volume of wastewater was 60 mL.

EC removed >99% of COD (COD_0_ of 307 ± 13 mg/L) by coagulation at a specific charge of 0.08 Ah/L, corresponding to a residence time of 10 min (Figure 4a). The EF process could also remove 88% of COD at 0.08 Ah/L, with 44% of COD decomposed by ^•^OH-mediated oxidation. Compared with EF, the overall COD removal efficiency of I-EF was lower due to the less contribution by coagulation. However, the ^•^OH-mediated oxidation of COD was equally pronounced, accounting for 44% at 0.17 Ah/L. Both EF and I-EF demonstrated superior overall COD removal efficiency than EO under the same test condition (Figure S6). Even though the EO process deployed NATO as the most prominent anode that outperformed other commercial electrodes in organic oxidation showcased in several previous studies,^24,41,42^ it could only remove >60% COD after 8 h (vs 20 min for I-EF and 10 min for EF). Coagulation gave EF and I-EF the leverage to outperform EO.

We evaluated the performance of EF and I-EF on removing low-concentration CMZ as a representative recalcitrant PPCPs pervasively present in municipal sewage and OWTS.^43,44^ The control test with EC showed no efficacy in removing CMZ (data not shown), while the EF and I-EF processes destroyed >50% of the CMZ within 10 min (Figure 4b). Though I-EF yielded more residual H_2_O_2_ in septic wastewater than EF (Figure S7a), the control test showed that the spiked H_2_O_2_ could not remove CMZ (Figure S8a). EF and I-EF with equivalent yields of ^•^OH are supposed to result in identical CMZ removal kinetics. However, I-EF demonstrated faster kinetics of CMZ removal. We validated that the IrO*_x_* anode could remove CMZ by direct oxidation (Figure S8b). Thus the accelerated CMZ removal in the I-EF could be attributed to the synergy with direct oxidation by the IrO*_x_* anode and the ^•^OH-mediated oxidation in the bulk solution, while EF only involved the latter pathway.

Both EF and I-EF release Fe^2+^ and Fe^3+^, which might be deemed burdens that increase process complexity. However, we would argue that these species facilitate the removal of phosphate via the formation of iron (II) phosphate, ferric orthophosphate, and the adsorption by Fe(OH)*_x_*.^45−47^
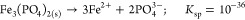
Rxn 6
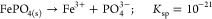
Rxn 7The EF process was applied to remove phosphate at high (25 mg/L) and low (5 mg/L) concentrations at an unamended initial pH of 5 in 10 mM NaClO_4_ (60 mL) electrolyte. As shown in Figure S9, a 5 min incubation period was observed, where flocs were under development and thus could not remove phosphate. After 10 min, EF reduced phosphate in both scenarios to below the discharge limit (1 mg/L phosphorus regulated by the USEPA, equivalent to 3 mg/L phosphate).^49^ The removal of phosphate by EF was further validated in treating septic wastewater containing 3 mg/L phosphate (Figure 4c). The higher initial pH (= 8) enables the rapid formation of flocs without an incubation period. Therefore, an instant removal of >99% phosphate was achieved after 2 min. The I-EF process exhibited equally fast kinetics in phosphate removal. It is concluded that I-EF and EF achieved simultaneous organic destruction and phosphate sequestration, which EO could not accomplish.

### Removal and Inactivation of Pathogens

The disinfection of gram-positive and negative bacteria by Fenton reactions has been extensively documented.^50,51^ This study provided new insights into EF and I-EF removal of bacteria and further expanded the investigation to virus inactivation, a much less explored aspect. *E. coli* and *E. durans* (or MS2 and Phi6) are spiked in sterilized septic wastewater at concentrations of 10^5^–10^6^ CFU/mL (or PFU/mL). The spiked bacteria concentrations are commensurate to the culturable bacteria concentration (2.4 × 10^4^ CFU/mL; Table S1) in raw septic water. The sterilization of wastewater enables the unbiased comparison of disinfection kinetics at similar starting concentrations.

**Figure 5:**
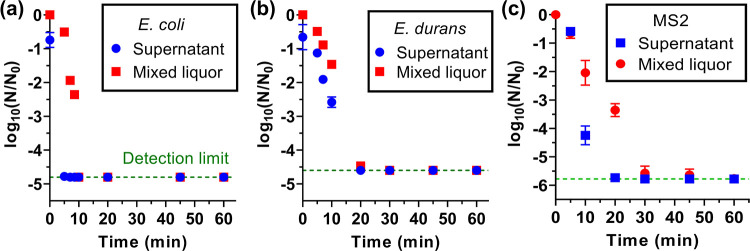
Removal and inactivation of (a) *E. coli* (gram-negative bacteria), (b) *E. durans* (gram-positive bacteria), and (c) MS2 non-enveloped bacteriophage by I-EF treatment of septic wastewater. Initial seeding concentrations for bacteria (or viruses) in 60 mL septic wastewater were 10^5^ – 10^6^ CFU/mL (or PFU/mL). A current of 30 mA was used in all the tests.

Pathogens could be inactivated by ^•^OH-mediated oxidation and separated by coagulation. Therefore, survived pathogens in mixed liquor and supernatant were analyzed to estimate the inactivation efficiency (by oxidation) and overall removal efficiency (oxidation and coagulation), respectively. For the first time, we demonstrate the side-by-side comparison of the disinfection performance of I-EF (Figure 5) and EF (Figure S10) operated at 30 mA.

In the I-EF processes, about 5-log removal of *E. coli* was achieved in 5 min; 5-log inactivation was achieved in 10 min (Figure 5a). The inactivation kinetics of the *E. durans* was slower. 5-log inactivation and removal required 20 min (Figure 5b). This is because gram-positive bacteria, such as *E. durans*, with a thick peptidoglycan cell wall, are more resistant to ^•^OH attack than gram-negative *E. coli*.^52^ I-EF also demonstrated capability in inactivating a non-enveloped virus, MS-2 (Figure 5c). The projected 5-log removal was achieved at 20 min. Though the inactivation lagged behind the apparent removal, 5-log inactivation was obtained at 30 min.

I-EF significantly outperformed EF in bacteria removal as the latter required >60 min to achieve 5-log removal. This is because I-EF yielded more H_2_O_2_ in septic wastewater (Figure S7b), which acted as a disinfectant. As for MS2 virus disinfection, I-EF and EF have similar kinetics. It is noteworthy that the removal and inactivation of Phi6, a model-enveloped virus to respiratory syndrome viruses,^32^ were validated in the EF process (Figure S10). A higher level of inactivation of Phi6 than MS2 (3.6 vs 2.4-log) was observed at 30 min, which is in agreement with the previous studies that enveloped viruses are more susceptible to structural damage by oxidation.^32,53^

A recent study reported the EC removal of MS2 and phi6, where 5-log removal required a long residence time of 100–120 min.^53^ As a comparison, EF and I-EF showed significantly faster virus removal and inactivation kinetics (residence time <60 min). This advantage could be attributed to the additional contribution of radical- and H_2_O_2_-mediated oxidation.

We previously conducted EO disinfection of latrine wastewater using a NATO anode. The 5-log inactivation of *E. coli* and MS2 was obtained at specific charges of 1.2 and 3 Ah/L, respectively.^24^ To meet the same disinfection goals, the charges of I-EF were 0.08 Ah/L (10 min for *E. coli*) and 0.25 Ah/L (30 min for MS2). The one order of magnitude lower charges indicates the higher disinfection performance of I-EF than EO.

### Formation of Byproducts

The discussion above demonstrates the versatile functionalities of EF and I-EF in organic removal (COD and trace contaminants), phosphate sequestration, and pathogen inactivation. More importantly, the fundamental investigation of radical chemistry indicates that chlorine and chlorine radicals were readily quenched by H_2_O_2_. This feature eliminates the generation of halogenated oxyanions and organics. We analyzed several typical disinfection byproducts (DBPs), including perchlorate, chlorate, THMs, and HAAs in treated septic wastewater after 10 min EF treatment (0.17 Ah/L), where COD and phosphate were completely removed. As shown in Table 1, the concentrations of THMs and HAAs in wastewater treated by EF were below the detection limit. No formation of chlorate or perchlorate was observed. The observations agree with the fundamental investigation above that the DBP precursors–free chlorine and chlorine radicals–were quenched by H_2_O_2_. Moreover, I-EF generated higher residual [H_2_O_2_] than EF throughout the treatment period (Figure S7), implying a stronger capability to quench chlorine species. Thus, the formation of DBPs in the I-EF process should also be negligible.

**Table 1:** Formation of Byproducts in Septic Wastewater Treatment by EF for 10 min and EO for 6 h to Achieve >50% COD Removal

byproducts	detection limit (μg/L)	EF (μg/L)	EO (μg/L)
chlorate	40	90,900 ± 381^a^	130,400 ± 550
perchlorate	40.0	ND^b^	2990.0 ± 20.0
*HAAs*			
monochloroacetic acid	20.0	ND	78.3 ± 51.9
dichloroacetic acid	10.00	ND	296 ± 129
trichloroacetic acid	1.00	ND	56.9 ± 40
dibromoacetic acid	1.0	ND	ND^b^
monobromoacetic acid	1.0	ND	ND^b^
*THMs*			
chloroform	0.24	ND	10.1 ± 7.55
bromodichloromethane	0.200	ND	ND^b^
bromoform	0.250	ND	ND^b^
dibromochloromethane	0.200	ND	ND^b^

a The septic wastewater has a background chlorate concentration of 91 mg/L. The EF treatment did not increase the chlorate concentration.

b Not detected due to the concentrations being below the detection limits.

As a comparison, EO treatment only removed 65% COD after 6 h (Figure S6). The analysis of DBPs in samples after 6 h of EO treatment showed the formation of mg/L-level chlorate and perchlorate, THMs, and HAAs (Table 1). Besides, EF and I-EF did not pose pH changes after treatment (Table S1). Overall, we concluded that EF and I-EF are “cleaner” alternatives to EO with higher water treatment performance and zero-formation of DBPs.

### Energy Consumption and Sludge Generation

**Figure 6:**
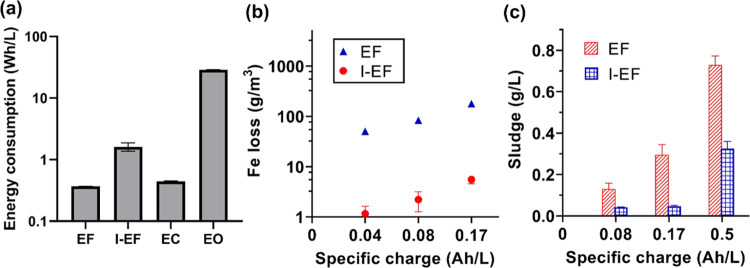
(a) Energy consumption of four electrochemical processes to remove >50% COD in septic wastewater. (b) Weight loss of LCS anode for EF and induced LCS plate for I-EF in 10 mM NaClO_4_ electrolyte. (c) Sludge generation in 10 mM NaClO_4_ electrolyte by EF and I-EF.

Despite the many advantages of EF and I-EF disclosed in this study, concerns about energy consumption and iron sludge management must be addressed. Figure 6a shows the energy consumption of treating septic wastewater by EF, I-EF, EC, and EO to achieve >50% COD removal. The EF process has the lowest energy consumption of 0.37 kWh/m^3^ at 0.08 Ah/L, which is ∼70 times smaller than EO and lower than those reported in centralized aerobic (1 kWh/m^3^) and anaerobic (0.43 kWh/m^3^) wastewater treatment processes in Europe^54−57^ and within the reported range (0.12–0.79 kWh/m^3^) in the United States.^58,59^

We estimated the mass loss of LCS anode per volume of water treated (g/L) by EF with respect to the specific charge by the Faradaic law and assuming the two-electron oxidation of Fe (Fe → Fe^2+^ + 2e^–^; *E*^0^ = 0.44 V_RHE_). The details of the calculation are described in Text S5. The mass loss was also measured experimentally by weighing the LCS plate before and after treatment (Figure 6b). As shown in Figure S11, the measured values aligned with the predicted values and were linearly correlated with the specific charges. Further, the faradaic efficiencies for the anodic Fe leaching were around 100%. This finding implies that the service life of the LCS anode can be determined by wastewater capacity and applied current.

We further quantified the dry weight of iron sludge (Text S6) produced by EF, which is linearly correlated with specific charges (Figure 6c). These data provide a critical design tool for EF-based on-site septic wastewater treatment systems. For instance, a family of three could produce wastewater at 680 L/day (assuming 60 gallons/person/day by USEPA^3^). Further, since the septic tank could remove *ca.* 80% of solid and the septic effluent usually contains 100 ± 50 mg/L total suspended solid,^3^ it can be concluded that the septic tank generates sludge at 273 g/day (= [100 mg/L/(1-0.8) −100 mg/L] × 680 L/day). If an EF reactor operated at 0.08 Ah/L (residence time of 10 min) is installed after the septic system, a >80% COD reduction and a >95% phosphate removal are expected. An extra 88 g/day of iron sludge will be generated by taking the 0.13 g/L sludge generation coefficient from Figure 6c. Although coupling the EF reactor with the septic system increases sludge production (i.e., 273 g/day of septic sludge and 88 g/day of iron-containing sludge), we believe this is not a deterring fact. Since the pumping and cleaning of a septic system is a common practice recommended every three to five years,^60^ the costs (e.g., $100–200 per service every two years based on the market price in northern New York) associated with the more frequent cleaning is marginal. Note that the sludge may contain particulate COD. The sludge should be collected with septic sludge for further disposal. Anaerobic sludge digestion could be an option because iron sludge as an additive can promote methane production.^61^

For treatment scenarios where minimum sludge production is intended, I-EF can be an alternative. The iron loss of the induced electrode in I-EF will be one order of magnitude slower than EF (Figure 6b), rendering a >50–80% reduction of sludge weight (Figure 6c).

## Conclusions

This study constructed highly efficient chemical-free EF and I-EF devices to realize one-step organic removal, pathogen inactivation, and phosphate sequestration in treating septic wastewater. The fundamental investigations combined probe molecule degradation and kinetic simulation to reveal that ^•^OH is the dominant radical species irrespective of the presence of Cl^–^. The EF and I-EF processes outperformed the conventional EO process in many aspects, including faster organic removal, zero byproduct formation, and lower energy consumption. Via theoretical calculation and experimental validation, insights into iron sludge management were provided. The overall results of this study imply that if EF or I-EF is adopted alone or as a post-treatment step after the septic tank, the discharge of various pollutants to the surface water could be largely minimized, which incurs profound impacts on many global environmental topics such as eutrophication mitigation, harmful algal bloom prevention, and waterborne pathogen transmission control.

## Supporting Information Available

Six text paragraphs, eleven figures, and two tables to support further discussion are included on the water physicochemical characteristics, pH and conductivity during phosphate removal, reaction kinetic modeling, evolution for [Fe^2+^] and [Fe^3+^], BA degradation; hydroxyl radicals’ concentration, radical speciation, and COD removal by EO (PDF)
