# Supplementary material for: Electro-Fenton and Induced Electro-Fenton as Versatile Wastewater Treatment Processes for Decontamination and Nutrient Removal without Byproduct Formation
Source: ACS ES T Eng. 2023 Jun 20;3(10):1547–56. doi: 10.1021/acsestengg.3c00128 (PMC10580281; doi:10.1021/acsestengg.3c00128)
Supplement: Supplementary file 1 — ee3c00128_si_001.pdf [file ee3c00128_si_001.pdf]

## *Supporting information*

**Electro-Fenton and induced electro-Fenton as versatile wastewater treatment processes for decontamination and nutrient removal without byproduct formation**

Luz Estefanny Quispe Cardenas,[a,b] Parker John Deptula,[a] Cynthia Soraya Huerta,[a] Chonglin Zhu,[c] Yinyin Ye,[c] Siwen Wang,[a]* Yang Yang [a]*

[a] Department of Civil and Environmental Engineering, Clarkson University

Potsdam, New York 13699 USA

[b] Institute for a Sustainable Environment, Clarkson University

Potsdam, New York 13699 USA

[c] Department of Civil, structural and environmental engineering, University at Buffalo

Buffalo, New York 14260 USA

*Corresponding Author:

Phone: +1 315-268-4446; Fax: +1 315-268-7985; E-mail: swang@clarkson.edu

Phone: +1 315-268-3861; Fax: +1 315-268-7985; E-mail: yanyang@clarkson.edu

**Text S1.** The calculation for COD removal by coagulation and oxidation

The COD removed by coagulation and oxidation was calculated based on the procedure and the equations below (Eq. S1 and S2), developed by Han *et al.* (2020)[1] with slight modifications,

$$\%\ COD_{by\ oxidation} = \frac{(COD_{raw\ wastewater} - COD_{mixed\ liquor})}{(COD_{raw\ wastewater})} \times 100\% \qquad \text{(Eq. S1)}$$

$$\%\ COD_{by\ coagulation} = \frac{(COD_{mixed\ liquor} - COD_{supernatant})}{(COD_{mixed\ liquor})} \times 100\% \qquad \text{(Eq. S2)}$$

where the mixed liquor is defined as the $Fe(OH)_x$-containing treated water. The supernatant was taken after centrifugal separation of $Fe(OH)_x$.

**Text S2.** Benzoic acid analysis

The benzoic acid analysis was performed using an ultra-high performance liquid chromatography (UHPLC) system (ExionLC 2.0+) coupled with a quadrupole time-of-flight mass spectrometer (QToF-MS) SCIEX 5600 Model X500B, USA). Separation was performed with an Atlantis® HILIC Silica column (3 μm, 2.1 × 100 mm). A linear gradient solvent program was operated with 5 mM ammonium acetate in LC-MS grade water (A) and acetonitrile (B). The gradient started from 90% B for a 1-min hold, then decreased to 50% over 1 min, and further decreased to 10% B from 2 to 2.4 min. The electrospray ionization (ESI) of the mass spectrometer was operated in the negative ion mode with a spray voltage of +4.5 kV and a capillary temperature of 350 °C. The collision energy was at 35 V, the declustering potential was at 60 V, and the default values were used for other parameters. Quantification was performed using multiple reaction monitoring (MRM), with MRM transition at m/z 50-1000 Da targeting the ionized compound (121.12 Da). The injection volume was set at 10 μL, and the oven temperature was fixed at 40 ℃. Data were acquired from Analyst 1.7 software and analyzed by PeakViews (AB SCIEX).

**Text S3.** Carbamazepine analysis

The analysis was performed using a UHPLC system (ExionLC 2.0+) coupled with a QToF-MS (SCIEX 5600 Model X500B, USA) operated in the electrospray ionization mode. Separation was performed with a C18 column (Gemini® 3 μm C18, 110 Å, 50 x 2 mm) purchased from Phenomenex (USA) at 40 ºC. An 11-min gradient elution, with a flow rate of 0.6 mL/min and an injection volume of 10 μL. The mobile phases of methanol and water were amended with 0.1% formic acid.

The data was acquired from Analyst 1.7 software using the high-resolution multiple reaction monitoring (HR-MRM) mode. The electrospray ionization (ESI) source conditions are as follows: spray voltage at + 5.5 kV, the capillary temperature at 350 °C, probe heater temperature at 430 °C, nebulize gas at 55 (GS2, arbitrary units), sheath gas at 50 (GS2, arbitrary units), curtain gas at 40 (arbitrary units), and sweep gas at 1 (arbitrary units), collision energy at 35 V, declustering potential at 100 V, other parameters were set by default.

**Text S4.** Pathogen cultivation and inactivation

*Preparation of bacterial stocks*

*Escherichia coli* (*E. coli* K-12 strain, ATCC 10798) and *Enterococcus durans* (*E. durans*, ATCC 6056) were selected as model gram-negative and gram-positive bacteria, respectively. The bacterial cultures were incubated overnight in Luria-Bertani (LB) Broth for *E. coli* and Brain Heart Infusion (BHI) Broth for *E. durans* at 37 °C in a shaking incubator at 180 rpm. Bacteria in the stationary phase were harvested by centrifugation at 5000 × g for 10 minutes, and the bacterial pellet was resuspended in phosphate-buffered saline (1 × PBS). The fresh bacterial stock was prepared for every experiment. The target concentration was $10^5$ – $10^6$ CFU/mL based on the culturable bacteria in the raw septic water (**Table S1**). The detection limit of 10 CFU/mL was determined following Sutton's method (2011).[2]

*Preparation of viral stocks*

Two bacteriophages were used as surrogates of pathogenic non-enveloped and enveloped human viruses: MS2 (ssRNA, non-enveloped, ATCC 15597-B1) and Phi6 (dsRNA, enveloped, provided by Dr. Ye Y. from University at Buffalo). LB agar and broth were used for MS2 propagation and its host (*Escherichia coli* C3000 [ATCC 15597]) cultivation at 37 °C. Tryptic soy agar (TSA) and broth were used for Phi6 propagation and its host (*Pseudomonas psyringae*) cultivation at 25 °C.

Liquid virus propagation and purification were performed for both viruses following Ye's protocols.[3] The supernatant, after centrifugation at 5000 × g for 10 min, was recovered and filtered. The filtrate was diluted 1000-fold after purification with a 0.2 µm syringe filter to obtain the final viral stock using 1 × PBS. The seeding level of both microorganisms in sterilized septic water was $10^5$ – $10^6$ PFU/mL, a typical value adopted by other disinfection studies.[4]

*Bacterial and viral inactivation and removal*

Bacteria and viruses were inoculated individually in the sterilized water in the electrochemical reactors. They were constantly mixed by a magnetic stirrer for 30 min before treatment.

The colony-counting and plaque assay were used to quantify viable bacteria and viruses, respectively, after overnight culture (16 ± 2 h).

The inactivation of bacteria and viruses from the treated septic water via coagulation or oxidation mechanisms is expressed in log reduction calculated by **equations S3** and **S4**. Mixed liquor refers to iron sludge-containing treated water without separation; the supernatant was obtained after 1 min centrifugation at 3500 rpm.

$$\text{Log reduction}_{\text{Inactivation}} = \text{Log}\left(\frac{\text{Bacteria titer}\left(\frac{CFU}{mL}\right)_{\text{raw wastewater}} - \text{Bacteria titer}\left(\frac{CFU}{mL}\right)_{\text{Mixed liquor}}}{\text{Bacteria titer}\left(\frac{CFU}{mL}\right)_{\text{raw liquor}}}\right) \quad \text{(Eq. S3)}$$

$$\text{Log reduction}_{\text{Removal}} = \text{Log}\left(\frac{\text{Bacteria titer}\left(\frac{CFU}{mL}\right)_{\text{raw wastewater}} - \text{Bacteria titer}\left(\frac{CFU}{mL}\right)_{\text{Supernatant}}}{\text{Bacteria titer}\left(\frac{CFU}{mL}\right)_{\text{raw liquor}}}\right) \quad \text{(Eq. S4)}$$

The results were expressed by $Log_{10}(N/N_0)$. The number of colonies is expressed on colony forming units (CFU) per mL of sample. As for virus analysis, the number of plaques is expressed in plaque-forming units (PFU) per mL.

**Text S5.** Theoretical mass loss of iron from LCS and Faradaic efficiency calculation

We assume that iron on LCS was oxidized and released as $Fe^{2+}$ ($Fe \rightarrow Fe^{2+} + 2e^-$). The theoretical mass loss of sacrificial LCS anode ($m_{Fe}$) in the EF processes was calculated based on Faraday's laws of electrolysis:

$$m_{Fe,\ theoretical} = \frac{I \times t \times M}{z \times F} \quad \text{(Eq. S5)}$$

where $I$ is the applied current (A), t is the reaction time (s), $M$ is the molar mass of the iron metal (55.8 g/mol), and $z$ = 2 is the number of electrons to oxidize Fe to $Fe^{2+}$. The Faraday constant ($F$) is 96,485 C/mol.

The Faradaic efficiency can then be calculated below

$$\text{Faradaic efficiency} = \frac{m_{Fe,\ measured}}{m_{Fe,\ theoretical}} \times 100\% \quad \text{(Eq. S6)}$$

where $m_{Fe,\ measured}$ is the mass loss of LCS determined by the weight difference before and after EF reactions.

**Text S6.** Iron sludge quantification

10 mL of treated mixed liquor generated from EF and I-EF in 10 mM $NaClO_4$ electrolyte was collected and filtrated using PTFE 0.45 µm membranes. The initial weight of the filters was then recorded. The filters with wet sludge were placed in the oven at 90 ºC overnight until the water was evaporated. The dry weight of the filter, together with the dry sludge, was recorded. The evaluation was performed at least in duplicates. The total sludge production was calculated as follows:

$$Sludge_{dry\ weight}\left(\frac{mg}{l}\right) = \left(\frac{(Filter + Sludge)_{Dry\ weight} - Filter_{initial\ weight}}{Mixed\ liquor\ (mL)}\right) \times 1000 \quad \text{(Eq. S7)}$$

**Table S1.** Septic wastewater composition before and after EF or EC treatment.

| | Septic wastewater | After EF_10 min | After EC_10 min |
|---|---|---|---|
| **pH** | 8.0 ± 0.10 | 8.4 ± 0.09 | 8.5 ± 0.03 |
| **Conductivity (μS/cm)** | 997.5 ± 32.5 | 907.6 ± 3.6 | 914.0 ± 2.8 |
| **COD (mg $O_2$/L)** | 307 ± 13 | < 3 [b] | < 3 |
| **$NH^{4+}$ (mg/L)** | 53.0 ± 2.5 | 47.0 ± 0.5 | 53.0 ± 2.5 |
| **Total phosphate (mg/L)** | 3.0 ± 0.81 | < 0.014 | < 0.014 |
| **$Cl^-$ (mg/L)** | 63.51 ± 1.07 | 57.40 ± 0.61 | 63.92 ± 0.76 |
| **Chlorate (mg/L)** | 91.2 ± 0.1 | 90.9 ± 3.8 | 91.8 ± 0.3 |
| **Culturable bacteria (CFU/mL)[a]** | $2.4 \times 10^4$ | - | - |

[a]: Culturable bacteria in raw septic tank wastewater were evaluated by cultivation in LB broth followed by colony counting. The procedure is the same as *E.coli* quantification described in **Text S4**.

[b]:"<" indicates the readings were below the detection limits.

**Table S2.** Reactions and rate constants involved in the EF reactions.

| Rxn No. | Reaction | Rate constant | Reference |
|---|---|---|---|
| | *ROS generation* | | |
| S1 | GDE → $H_2O_2$ | $2.0 \times 10^{-6}$ $s^{-1}$ for 30 mA | Fitted |
| | | $4.0 \times 10^{-6}$ $s^{-1}$ for 60 mA | 30 mA value × 2 |
| S2 | $H_2O_2$ → 2HO• | $5.0 \times 10^{-3}$ $s^{-1}$; EF at 30 mA | Fitted |
| | | $1.0 \times 10^{-2}$ $s^{-1}$; EF at 60 mA | Constant at 30 mA × 2 |
| | | $2.2 \times 10^{-3}$ $s^{-1}$; I-EF at 30 mA | Fitted |
| | | $4.4\times 10^{-3}$ $s^{-1}$; I-EF at 60 mA | Constant at 30 mA × 2 |
| | *pH-dependent equilibrium* | | |
| S3 | $H^+ + OH^- \rightarrow H_2O$ | $1.00 \times 10^{11}$ $M^{-1}$ $s^{-1}$ | [5] |
| S4 | $H_2O \rightarrow H^+ + OH^-$ | $1.00 \times 10^{-3}$ $s^{-1}$ | [5] |
| S5 | $OCl^- + H^+ \rightarrow HOCl$ | $5.00 \times 10^{10}$ $M^{-1}$ $s^{-1}$ | [5] |
| S6 | $HOCl \rightarrow OCl^- + H^+$ | $1.60 \times 10^{3}$ $s^{-1}$ | [5] |
| S7 | $H_2O_2 \rightarrow H^+ + HO_2^-$ | 1.00 $s^{-1}$ | From $pK_a$ |
| S8 | $H^+ + HO_2^- \rightarrow H_2O_2$ | $1.60 \times 10^{12}$ $M^{-1}$ $s^{-1}$ | From $pK_a$ |
| | Cl• generation | | |
| S9 | $Cl^-$ + HO• → ClOH• | $4.30 \times 10^{9}$ $M^{-1}$ $s^{-1}$ | [6] |
| S10 | ClOH• → $Cl^-$ + HO• | $6.10 \times 10^{9}$ $s^{-1}$ | [7] |
| S11 | Cl• + $OH^-$ → $ClOH•^-$ | $1.80 \times 10^{10}$ $M^{-1}$ $s^{-1}$ | [8] |
| S12 | $ClOH•^-$ + $H^+$ → Cl• + $H_2O$ | $2.10 \times 10^{10}$ $M^{-1}$ $s^{-1}$ | [7] |
| S13 | $ClOH•^-$ + $Cl^-$ → $Cl_2•^-$ + $OH^-$ | $1.00 \times 10^{5}$ $M^{-1}$ $s^{-1}$ | [9] |
| S14 | $Cl_2•^-$ + $OH^-$ → $ClOH•^-$ + $Cl^-$ | $4.50 \times 10^{7}$ $M^{-1}$ $s^{-1}$ | [9] |
| S15 | Cl• + $Cl^-$ → $Cl_2•^-$ | $6.50 \times 10^{9}$ $M^{-1}$ $s^{-1}$ | [8] |
| S16 | $Cl_2•^-$ → Cl• + $Cl^-$ | $1.10 \times 10^{5}$ $s^{-1}$ | [7] |
| | *$Cl_2$ generation via radical pathways* | | |
| S17 | Cl• + Cl• → $Cl_2$ | $1.00 \times 10^{8}$ $M^{-1}$ $s^{-1}$ | [10] |
| S18 | Cl• + $Cl_2•^-$ → $Cl^-$ + $Cl_2$ | $1.40 \times 10^{9}$ $M^{-1}$ $s^{-1}$ | [11] |

| | | | |
|---|---|---|---|
| S19 | $Cl_2•^- + Cl_2•^- \rightarrow 2Cl^- + Cl_2$ | $8.30 \times 10^8$ $M^{-1}$ $s^{-1}$ | 6 |
| S20 | $Cl_2•^- + HO• \rightarrow HOCl + Cl^-$ | $1.00 \times 10^9$ $M^{-1}$ $s^{-1}$ | 6 |
| | *$Cl_2$ dissolution* | | |
| S21 | $Cl_2 + H_2O \rightarrow Cl_2OH^- + H^+$ | $1.50 \times 10^1$ $M^{-1}$ $s^{-1}$ | 12 |
| S22 | $Cl_2OH^- \rightarrow HOCl + Cl^-$ | $5.50 \times 10^9$ $s^{-1}$ | 12 |
| | *HO• consumption* | | |
| S23 | $HO• + HO• \rightarrow H_2O_2$ | $5.50 \times 10^9$ $s^{-1}$ | |
| | *HO• transformation* | | |
| S24 | $HO• \rightarrow O•^- + H^+$ | $1.26 \times 10^{12}$ $s^{-1}$ | 13 |
| S25 | $O•^- + H_2O \rightarrow HO• + OH^-$ | $1.80 \times 10^6$ $M^{-1}$ $s^{-1}$ | 13 |
| S26 | $HO• + OH^- \rightarrow O•^- + H_2O$ | $1.30 \times 10^{10}$ $M^{-1}$ $s^{-1}$ | 13 |
| S27 | HO• → Products | $2.70 \times 10^7$ $s^{-1}$ for EF | Fitted value[a] |
| | | $2.20 \times 10^7$ $s^{-1}$ for I-EF | Fitted value |
| | *$HO_2^-$, $HO_2•$, $O_2•^-$ related* | | |
| S28 | $HO• + O•^- \rightarrow HO_2^-$ | $1.00 \times 10^{10}$ $M^{-1}$ $s^{-1}$ | 13 |
| S29 | $HO• + HO_2^- \rightarrow HO_2• + OH^-$ | $7.50 \times 10^9$ $M^{-1}$ $s^{-1}$ | 13 |
| S30 | $HO_2• + O_2•^- \rightarrow HO_2^- + O_2$ | $9.70 \times 10^7$ $M^{-1}$ $s^{-1}$ | 13 |
| S31 | $HO• + HO_2• \rightarrow H_2O + O_2$ | $6.60 \times 10^9$ $M^{-1}$ $s^{-1}$ | 13 |
| S32 | $HO_2• + HO_2• \rightarrow H_2O_2 + O_2$ | $8.30 \times 10^5$ $M^{-1}$ $s^{-1}$ | 13 |
| S33 | $HO_2• \rightarrow H^+ + O_2•^-$ | $1.60 \times 10^5$ $s^{-1}$ | 14 |
| S34 | $HO• + O_2•^- \rightarrow OH^- + O_2$ | $8.00 \times 10^9$ $M^{-1}$ $s^{-1}$ | 13 |
| | *Radicals quenched by free chlorine* | | |
| S35 | $HO• + HOCl \rightarrow ClO• + H_2O$ | $2.00 \times 10^9$ $M^{-1}$ $s^{-1}$ | 5 |
| S36 | $HO• + OCl^- \rightarrow ClO• + OH^-$ | $8.80 \times 10^9$ $M^{-1}$ $s^{-1}$ | 15 |
| S37 | $Cl• + HOCl \rightarrow ClO• + H^+ + Cl^-$ | $3.00 \times 10^9$ $M^{-1}$ $s^{-1}$ | 16 |
| S38 | $Cl• + OCl^- \rightarrow ClO• + Cl^-$ | $8.20 \times 10^9$ $M^{-1}$ $s^{-1}$ | 7 |
| | *Radicals quenched by benzoic acid* | | |
| S39 | $HO• + C_6H_5COO^- \rightarrow$ Products | $5.90 \times 10^9$ $M^{-1}$ $s^{-1}$ | 13 |
| S40 | $Cl• + C_6H_5COO^- \rightarrow$ Products | $1.80 \times 10^{10}$ $M^{-1}$ $s^{-1}$ | 17 |
| S41 | $Cl_2•^- + C_6H_5COO^- \rightarrow$ Products | $2.00 \times 10^6$ $M^{-1}$ $s^{-1}$ | 18 |

| | | | |
|---|---|---|---|
| S42 | $O^{\bullet-} + C_6H_5COO^- \rightarrow$ Products | $4.00 \times 10^7$ $M^{-1}$ $s^{-1}$ | [13] |
| | *Radicals and free chlorine quenched by $H_2O_2$* | | |
| S43 | $HO^\bullet + H_2O_2 \rightarrow HO_2^\bullet + H_2O$ | $2.70 \times 10^7$ $M^{-1}$ $s^{-1}$ | [13] |
| S44 | $Cl^\bullet + H_2O_2 \rightarrow HO_2^\bullet + Cl^- + H^+$ | $2.00 \times 10^9$ $M^{-1}$ $s^{-1}$ | [19] |
| S45 | $HOCl + H_2O_2 \rightarrow H^+ + Cl^- + H_2O + O_2$ | $1.10 \times 10^4$ $M^{-1}$ $s^{-1}$ | [15] |
| S46 | $OCl^- + H_2O_2 \rightarrow Cl^- + H_2O + O_2$ | $1.70 \times 10^5$ $M^{-1}$ $s^{-1}$ | [15] |
| | Electrochemical chlorine evolution | | |
| S47 | $Cl^- + H_2O \rightarrow OCl^- + 2e^- + 2H^+$ | $1 \times 10^{-4}$ $M^{-1}$ $s^{-1}$ | Fitted value at 30 mA [b] |

[a] Rxn S27 stands for the unknown HO• quenching pathways, in addition to the pathways in which •OH reacts with HO•, $H_2O_2$, chlorine, and $Cl^-$. The rate constant of rxn 27 was arbitrarily set as a value equal to or smaller than rxn 43, as we believe the unknown quenching reactions should not be faster than the homogeneous reaction between HO• and $H_2O_2$.

[b] Rxn S47 refers to the electrochemical oxidation of $Cl^-$ to $OCl^-$ by IrOx anode in the HPP mode following the higher oxide oxidation pathway.[20] The rate constant was obtained by fitting the experimental data in **Figure S3a**.

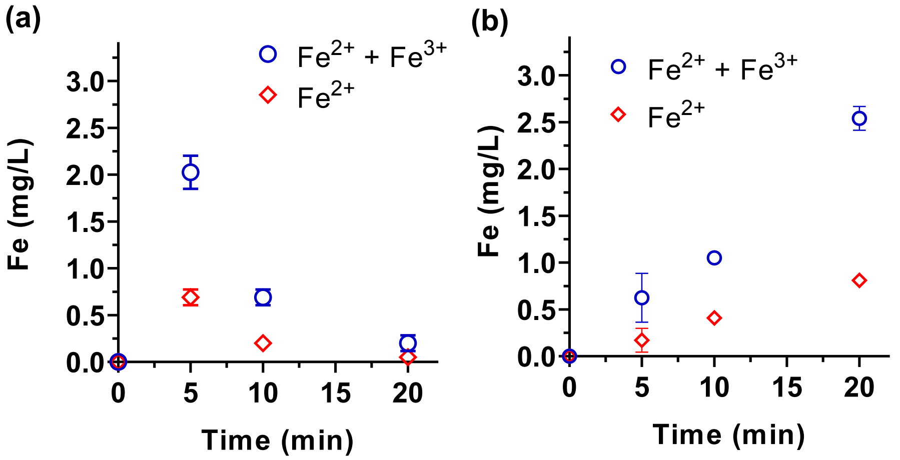


**Figure S1.** Evolution of soluble [$Fe^{2+}$] and [$Fe^{3+}$] of EF process in (a) 10 mM $NaClO_4$ electrolyte and (b) septic wastewater at 30 mA. The pH values of electrolyte and wastewater are 6.5 and 8.0, respectively.

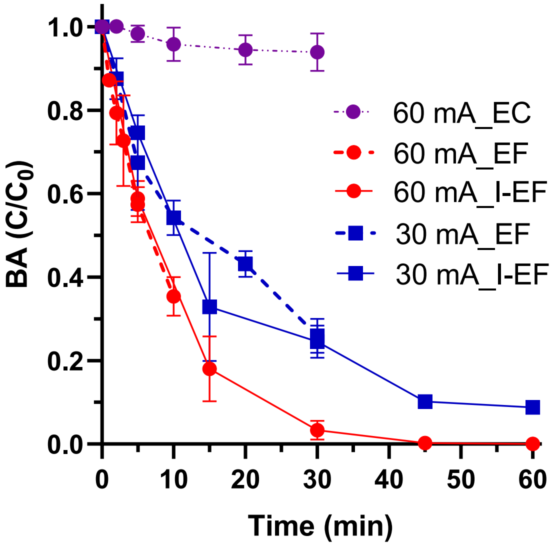


**Figure S2**. Removal of 1 mM BA in 10 mM $NaClO_4$ (60 mL; pH = 4.0) by EC, EF, and I-EF processes.

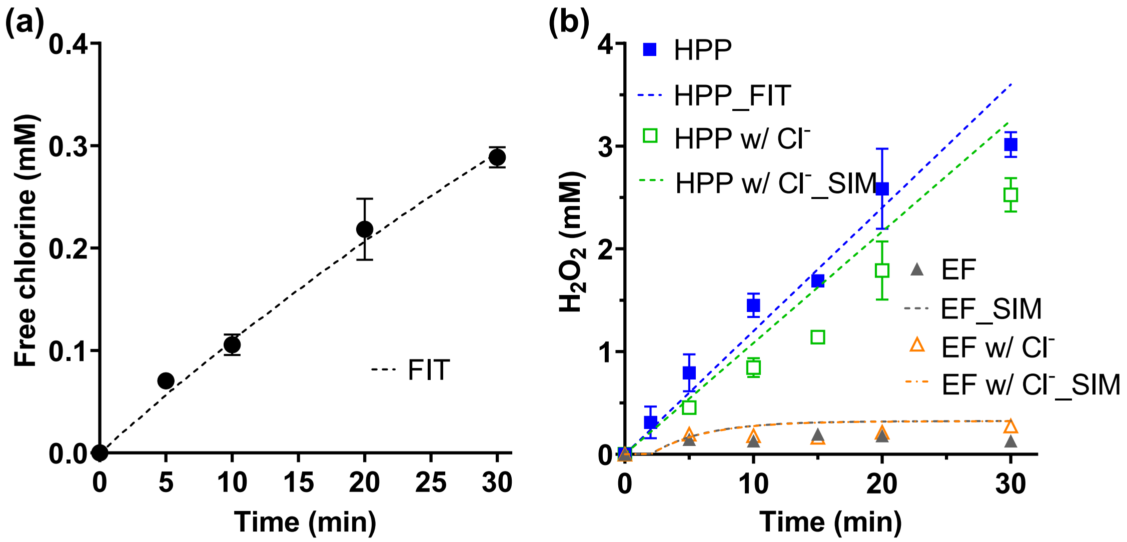


**Figure S3.** (a) Chlorine evolution by $IrO_x$ anode in 1.8 mM NaCl + 10 mM $NaClO_4$ electrolyte. The $IrO_x$ anode (6 $cm^2$) was paired with an SS cathode (6 $cm^2$) and operated at 30 mA. The data were used to calibrate the **Rxn S47** of **Table S2**. (b) $H_2O_2$ evolution profiles in HPP and EF modes with or without $Cl^-$ at operated 30 mA. Dots are experimental data, while lines are modeling results. The data set denoted by "FIT" means the data were fed to the kinetic models to calibrate specific rate constants; those tagged as "SIM" are results predicted by the calibrated kinetic models without manual intervention. Lines of "EF_SIM" and "EF w/Cl-_SIM" overlapped because the model simulation concluded that $Cl^-$ did not impact $H_2O_2$ evolution.

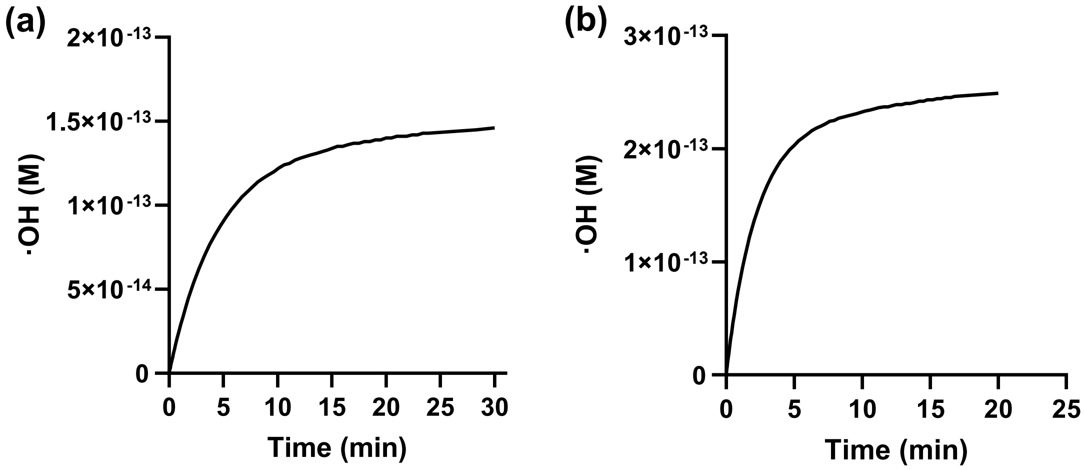


**Figure S4.** Hydroxyl radical concentrations as a function of EF reaction time predicted by the model simulation at (a) 30 mA and (b) 60 mA.

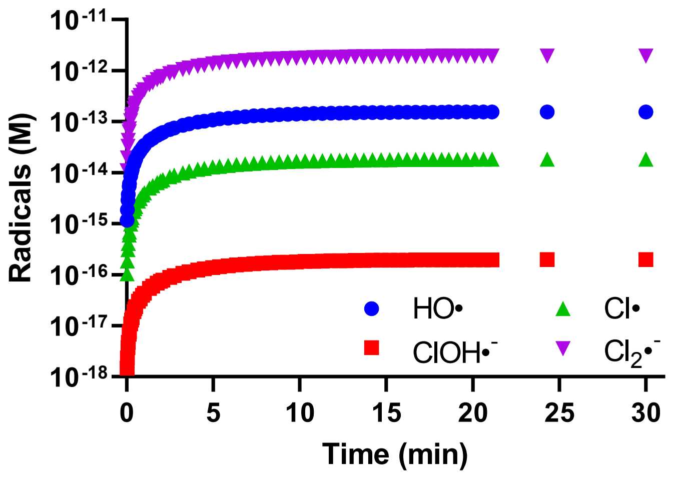


**Figure S5**. The radical speciation when 1.8 mM $Cl^-$ in the EF reaction predicted by the model excluding Rxn S44.

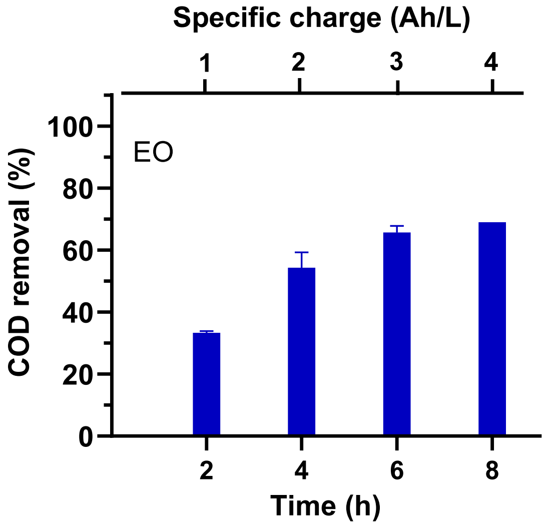


**Figure S6**. COD removal by EO using a NATO anode paired with a SS cathode. A current of 30 mA was applied on 6 $cm^2$ electrodes to treat 60 mL septic wastewater.

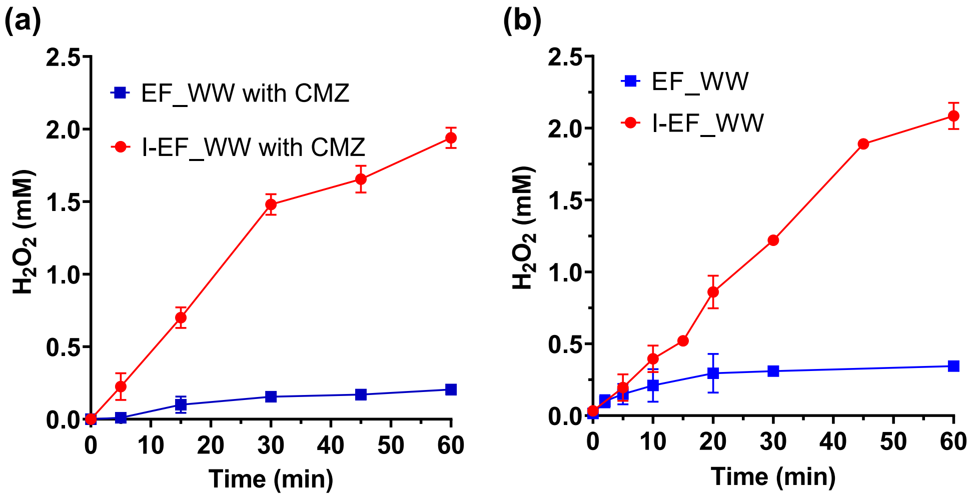


**Figure S7**. Evolution of $H_2O_2$ during the treatment of septic wastewater (a) with or (b) without CMZ (1 μM) by EF and I-EF at 30 mA. Wastewater amended with CMZ was used in the investigation of organic removal efficiency. Raw wastewater was used in disinfection studies. It can be concluded that the trace amount of CMZ did not impact $H_2O_2$ evolution.

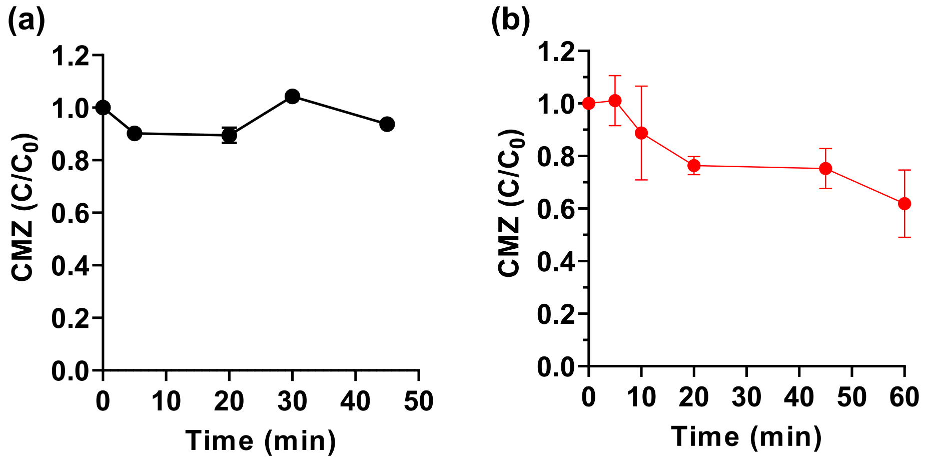


**Figure S8.** Removal of carbamazepine (CMZ; $C_0$ = 1 μM) in (a) septic wastewater by spiking 2 mM $H_2O_2$ and (b) in 10 mM $NaSO_4$ by electrolysis using an $IrO_x$ anode (6 $cm^2$) coupled with a SS cathode (6 $cm^2$) at 30 mA. The anodic potential of $IrO_x$ was ~1.5 $V_{RHE,}$ which is insufficient for •OH generation ($E^0$ = 2.7 $V_{RHE}$), leaving direct oxidation the only possible pathway that contributed to CMZ removal. The direct oxidation should still be a pronounced pathway when the $IrO_x$ anode was incorporated with an induced LCS plate and a GDE cathode in the I-EF process at 30 mA.

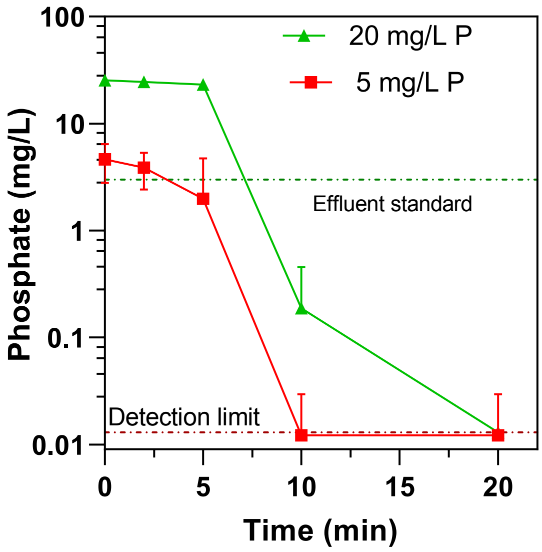


**Figure S9**. Phosphate sequestration in 10 mM $NaClO_4$ (60 mL) electrolyte by EF treatment at 30 mA.

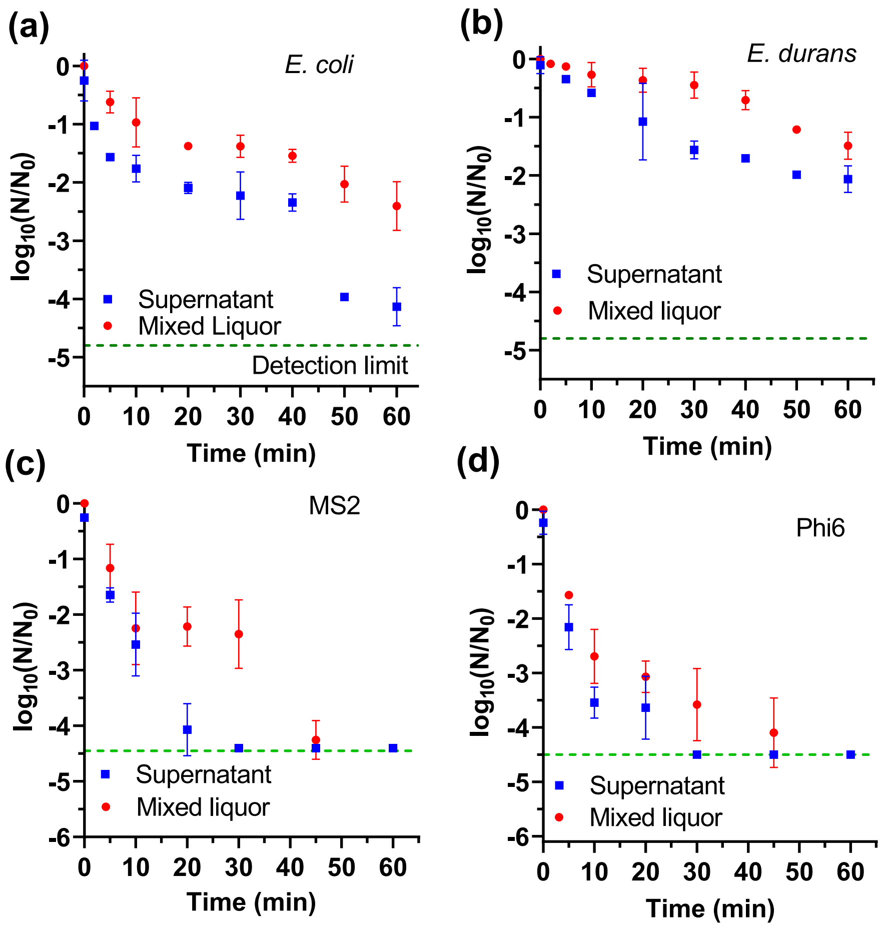


**Figure S10.** Removal and inactivation of (a) *Escherichia coli* (gram-negative bacteria), (b) *Enterococcus durans* (gram-positive bacteria), (c) MS2 (non-enveloped bacteriophage), and (d) Phi6 (enveloped bacteriophage) in the EF treatment of septic wastewater. Initial seeding concentrations for bacteria (or viruses) were $10^5 - 10^6$ CFU/mL (or PFU/mL). A current of 30 mA was used in all the tests.

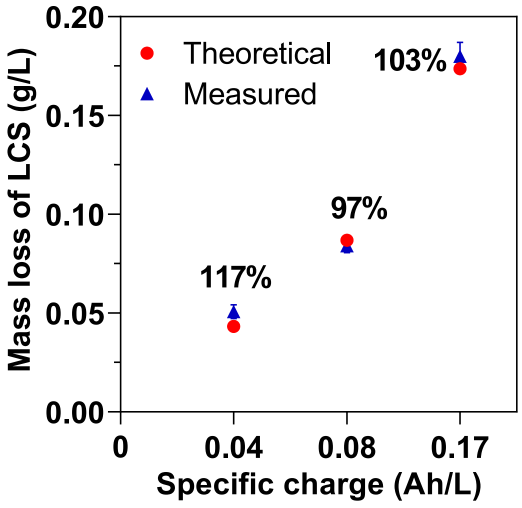


**Figure S11**. Mass loss of LCS anode as a function of specific charge estimated by the Faradaic law and measured by weighing. The percentage values represent the Faradaic efficiencies of the anodic corrosion of LCS ($Fe \rightarrow Fe^{2+} + 2e^{-}$), which were calculated by Eq. S6 in **Text S5**.

## References


(1) Han, X.; Lu, H.; Gao, Y.; Chen, X.; Yang, M. The Role of in Situ Fenton Coagulation on the Removal of Benzoic Acid. *Chemosphere* **2020**, *238*, 124632. https://doi.org/10.1016/j.chemosphere.2019.124632.

(2) Sutton, S. Accuracy of Plate Counts. *J. Valid. Technol.* **2011**, 5.

(3) Ye, Y.; Chang, P. H.; Hartert, J.; Wigginton, K. R. Reactivity of Enveloped Virus Genome, Proteins, and Lipids with Free Chlorine and UV254. *Environ. Sci. Technol.* **2018**, *52* (14), 7698–7708. https://doi.org/10.1021/acs.est.8b00824.

(4) Huang, X.; Qu, Y.; Cid, C. A.; Finke, C.; Hoffmann, M. R.; Lim, K.; Jiang, S. C. Electrochemical Disinfection of Toilet Wastewater Using Wastewater Electrolysis Cell. *Water Res.* **2016**, *92*, 164–172. https://doi.org/10.1016/j.watres.2016.01.040.

(5) Matthew, B. M.; Anastasio, C. A Chemical Probe Technique for the Determination of Reactive Halogen Species in Aqueous Solution: Part 1 – Bromide Solutions. *Atmospheric Chem. Phys.* **2006**, *6* (9), 2423–2437. https://doi.org/10.5194/acp-6-2423-2006.

(6) *NDRL/NIST Solution Kinetics Database*. https://kinetics.nist.gov/solution/ (accessed 2019-09-20).

(7) G. Jayson, G.; J. Parsons, B.; J. Swallow, A. Some Simple, Highly Reactive, Inorganic Chlorine Derivatives in Aqueous Solution. Their Formation Using Pulses of Radiation and Their Role in the Mechanism of the Fricke Dosimeter. *J. Chem. Soc. Faraday Trans. 1 Phys. Chem. Condens. Phases* **1973**, *69* (0), 1597–1607. https://doi.org/10.1039/F19736901597.

(8) Kläning, U. K.; Wolff, T. Laser Flash Photolysis of HClO, ClO−, HBrO, and BrO− in Aqueous Solution. Reactions of Cl- and Br-Atoms. *Berichte Bunsenges. Für Phys. Chem.* **1985**, *89* (3), 243–245. https://doi.org/10.1002/bbpc.19850890309.

(9) Grebel, J. E.; Pignatello, J. J.; Mitch, W. A. Effect of Halide Ions and Carbonates on Organic Contaminant Degradation by Hydroxyl Radical-Based Advanced Oxidation Processes in Saline Waters. *Environ. Sci. Technol.* **2010**, *44* (17), 6822–6828. https://doi.org/10.1021/es1010225.

(10) Wu, D.; Wong, D.; Di Bartolo, B. Evolution of Cl−2 in Aqueous NaCl Solutions. *J. Photochem.* **1980**, *14* (4), 303–310. https://doi.org/10.1016/0047-2670(80)85102-1.

(11) Park, H.; Vecitis, C. D.; Hoffmann, M. R. Electrochemical Water Splitting Coupled with Organic Compound Oxidation: The Role of Active Chlorine Species. *J. Phys. Chem. C* **2009**, *113* (18), 7935–7945. https://doi.org/10.1021/jp810331w.

(12) Wang, T. X.; Margerum, D. W. Kinetics of Reversible Chlorine Hydrolysis: Temperature Dependence and General-Acid/Base-Assisted Mechanisms. *Inorg. Chem.* **1994**, *33* (6), 1050–1055. https://doi.org/10.1021/ic00084a014.

(13) Buxton, G. V.; Greenstock, C. L.; Helman, W. P.; Ross, A. B. Critical Review of Rate Constants for Reactions of Hydrated Electrons, Hydrogen Atoms and Hydroxyl Radicals (·OH/·O− in Aqueous Solution. *J. Phys. Chem. Ref. Data* **1988**, *17* (2), 513–886. https://doi.org/10.1063/1.555805.

(14) Bielski, B. H. J.; Cabelli, D. E.; Arudi, R. L.; Ross, A. B. Reactivity of HO2/O−2 Radicals in Aqueous Solution. *J. Phys. Chem. Ref. Data* **1985**, *14* (4), 1041–1100. https://doi.org/10.1063/1.555739.

(15) Connick, R. E. The Interaction of Hydrogen Peroxide and Hypochlorous Acid in Acidic Solutions Containing Chloride Ion. *J. Am. Chem. Soc.* **1947**, *69* (6), 1509–1514. https://doi.org/10.1021/ja01198a074.

(16) Zehavi, D.; Rabani, J. Oxidation of Aqueous Bromide Ions by Hydroxyl Radicals. Pulse Radiolytic Investigation. *J. Phys. Chem.* **1972**, *76* (3), 312–319. https://doi.org/10.1021/j100647a006.

(17) Mártire, D. O.; Rosso, J. A.; Bertolotti, S.; Le Roux, G. C.; Braun, A. M.; Gonzalez, M. C. Kinetic Study of the Reactions of Chlorine Atoms and Cl2•- Radical Anions in Aqueous Solutions. II. Toluene, Benzoic Acid, and Chlorobenzene. *J. Phys. Chem. A* **2001**, *105* (22), 5385–5392. https://doi.org/10.1021/jp004630z.

(18) Hasegawa, K.; Neta, P. Rate Constants and Mechanisms of Reaction of Chloride (Cl2-) Radicals. *J. Phys. Chem.* **1978**, *82* (8), 854–857. https://doi.org/10.1021/j100497a003.

(19) Yu, X.-Y. Critical Evaluation of Rate Constants and Equilibrium Constants of Hydrogen Peroxide Photolysis in Acidic Aqueous Solutions Containing Chloride Ions. *J. Phys. Chem. Ref. Data* **2004**, *33* (3), 747–763. https://doi.org/10.1063/1.1695414.

(20) Trasatti, S. Progress in the Understanding of the Mechanism of Chlorine Evolution at Oxide Electrodes. *Electrochimica Acta* **1987**, *32* (3), 369–382. https://doi.org/10.1016/0013-4686(87)85001-6.
